# Molecular and physiological analysis of *Anopheles funestus* swarms in Nchelenge, Zambia

**DOI:** 10.1186/s12936-018-2196-6

**Published:** 2018-01-25

**Authors:** Jacek W. Zawada, Yael L. Dahan-Moss, Mbanga Muleba, Roch K. Dabire, Hamid Maïga, Nelius Venter, Craig Davies, Richard H. Hunt, Maureen Coetzee, Lizette L. Koekemoer

**Affiliations:** 1 Wits Research Institute for Malaria; School of Pathology, Faculty of Health Sciences, University of the Witwatersrand, Johannesburg, South Africa; 2 Center for Opportunistic, Tropical and Hospital Infections, National Institute for Communicable Diseases, Sandringham, Johannesburg, South Africa; 3 Tropical Diseases Research Centre, Ndola, Zambia; 4 Institut de Recherche en Sciences de la Santé/Centre Muraz, BP 545, Bobo-Dioulasso, Burkina Faso

**Keywords:** Genitalia rotation, Male biology, Restriction fragment length polymorphism, Clade analysis, Swarming

## Abstract

**Background:**

*Anopheles funestus* has been recognized as a major malaria vector in Africa for over 100 years, but knowledge on many aspects of the biology of this species is still lacking. *Anopheles funestus*, as with most other anophelines, mate through swarming. A key event that is crucial for the *An. funestus* male to mate is genitalia rotation. This involves the 135° to 180° rotation of claspers, which are tipped with claws. This physical change then enables the male to grasp the female during copulation. The aim of this investigation was to molecularly characterize wild *An. funestus* swarms from Zambia and examine the degree of genitalia rotation within the swarm.

**Methods:**

*Anopheles funestus* swarms were collected from Nchelenge, northern Zambia, during dusk periods in May 2016. All the adults from the swarm were analysed morphologically and identified to species level using a multiplex PCR assay. *Anopheles funestus s.s.* specimens were molecularly characterized by restriction fragment length polymorphism type and Clade type assays. The different stages of genitalia rotation were examined in the adult males.

**Results:**

A total of six swarms were observed during the study period and between 6 and 26 mosquitoes were caught from each swarm. Species analysis revealed that 90% of the males from the swarms were *An. funestus s.s.* MW-type, with 84% belonging to clade I compared to 14% clade II and 2% failed to amplify. Very few specimens (3.4%) were identified as *Anopheles gambiae s.s*. Eighty percent of the males from the swarm had complete genitalia rotation.

**Conclusions:**

This is the first time that *An. funestus* swarms have been molecularly identified to species level. *Anopheles funestus* swarms appear to be species-specific with no evidence of clade-type differentiation within these swarms. The *An. funestus* swarms consist mainly of males with fully rotated genitalia, which strongly suggests that swarming behaviour is triggered primarily when males have matured.

## Background

The latest WHO World Malaria Report states that approximately 429,000 malaria deaths (range 235,000–639,000) occurred in 2015, of which 92% of these were in Africa [1]. Malaria vector species in Africa occur mainly in two taxonomic groupings: the *Anopheles funestus* group and the *Anopheles gambiae* complex. The *An. funestus* group is broadly distributed across the African continent, with 11 recognized species: *An. funestus*, *An. funestus*-like, *Anopheles vaneedeni*, *Anopheles leesoni*, *Anopheles rivulorum*, *An. rivulorum*-like, *Anopheles parensis*, *Anopheles fuscivenosus*, *Anopheles aruni*, *Anopheles brucei*, and *Anopheles confusus* [2–6]. *Anopheles funestus* sensu stricto (s.s.) is a major vector of malaria in Africa due to its efficiency in transmitting *Plasmodium* and its anthropophilic and endophilic feeding and resting behaviours [2, 3]. *Anopheles funestus* is widely distributed over tropical and subtropical Africa where it breeds in permanent water bodies [2, 3].

Molecular studies have shown genetic structuring in populations of *An. funestus* using restriction fragment length polymorphism (RFLP) genotyping [7, 8] as well as mitochondrial clade analysis [9, 10]. The RFLP typing differentiates the population in mainly M-, W- and MW-type populations and is associated with the geographical distribution of the species. M-type is found in *An. funestus* populations located on the East side of the Rift valley and has been reported in Madagascar, Kenya and Mali [8]. The MW-type was reported from Angola, Malawi, South Africa and Mozambique. The W-type is widespread in Africa and was reported on the west side of the Rift valley spanning into Kenya [8]. Mitochondrial analysis report similar population structuring as found with RFLP analysis [9].

Population structuring using mitochondrial analysis placed *An. funestus* into two clades: clade I or clade II [9, 10]. Clade I was recorded widespread from 12 African countries, clade II in combination with clade I, has only been reported from Zambia, Mozambique and Madagascar [9–11]. The proportion of clade I: clade II between the countries vary and Zambia showed a ratio of 80% clade I and 20% of population belong to clade II [11], Mozambique and Madagascar populations are different with 60% of the population belonging to clade II, while remaining 40% belong to clade I [10, 11].

*Anopheles funestus,* as with other anophelines, mate via creating swarms. *Anopheles funestus* swarms, occur at sunset in relatively open areas [12]. Studies by Harper [13] observed that each *An. funestus* swarm is generally of a similar size and these swarms reform on subsequent nights in the same location. Harper [13] observed *An. funestus* swarming a foot (0.3 m) above the ground and within the entrance of huts in East Africa. In contrast, Charlwood et al. [12] established that *An. funestus* from Mozambique swarm 2–4 m above the ground and well away from houses. Harper [13] and Charlwood et al. [12] unfortunately did not confirm the species identity using molecular identification [14], which might explain the difference in swarm height observed between these two studies.

*Anopheles funestus* swarm formation begins when one or more males start flying simultaneously, then the swarm numbers rapidly increase reaching a maximum size and density within 5 min [12]. Subsequently, a female enters the swarm and mate with one of the swarming males [15, 16]. Swarm observations have clearly showed that females will choose a specific mate [15, 16]. The harmonization of wing beat frequencies between males and females appears to play a role in successful mating [17] amongst others. Since it was unclear if young sexual immature males participate in swarming, it was impossible to predict if females use male maturity to differentiate between potential mates.

A hall mark event in the sexual maturity of the *An. funestus* male is genitalia rotation [16, 18]. Male genitalia rotation event involves the abdominal segments 8 through 10, which include claspers that are tipped with claws [18, 19]. These claspers need to be rotated by 135°–180° for them to be able to grasp the female during copulation [16, 18]. Dahan and Koekemoer [19] showed that genitalia rotation of *An. funestus* reaches its full rotation stage between 14 and 36 h post-emergence at 23 ± 1 °C. Furthermore, there was no difference in the rate of genitalia rotation between laboratory colonized and wild *An. funestus* males reared under controlled conditions [19].

It is well known that genitalia rotation is essential for mating to take place [20]. This automatically deduce the conclusion that newly emerged or young males will not participate in swarms in order to reserve their energy reserves until they are physically able to find a mate [15, 21–23]. Swarming of young immature males will also expose them unnecessarily to the threat to predation [24].

Although numerous papers report on swarming in various different Dipterans, the physical state or stage of genitalia rotation of swarming males have not been recorded. No published literature could be found to confirm that young immature males are not joining in swarming activities.

Studies by Harper, Charlwood, Dahan and Koekemoer, Dahan-Moss and Koekemoer [12, 13, 19, 25] have significantly contributed to the knowledge on *An. funestus* male mosquito maturation, mating and swarming. However, the underlying molecular and physiological characteristics of the adult *An. funestus* that form a swarm are still limited.

In this study, wild *An. funestus* swarms were investigated, and for the first time their species identity was confirmed as well as the stage of male genitalia rotation. In addition to this, molecular markers previously used to differentiate population substructure were applied to samples collected from wild caught swarms.

## Methods

### Biological material and swarm collection

Prior to field work, preservation of samples was optimized at the National Institute for Communicable Diseases using laboratory reared *An. funestus* (FUMOZ) samples. This was done to ensure that the preservation method did not affect the collected material and that post-collection analysis could still be performed. Freshly collected samples were analysed and genitalia rotation scored before placing them into three different preservation media (silica or ethanol or RNAlater) for 72 h. The genitalia rotation of the samples were then re-analysed and compared with their original score. Analysis revealed that preservation did not interfere with interpretation of results. Although laboratory controlled environment in Johannesburg, showed that any of the three preservation methods could be used, samples were stored in either ethanol or on silica.

The biological material was collected using sweep nets from 18 to 25 May 2016, in Nchelenge, northern Zambia (S09°19′28.6″ E028°47′06.9″), during the dusk period every day (18:00–18:30 pm) which is the best time to collect maximum numbers of adult mosquitoes [12, 26]. The swarms were caught near a dwelling, in a village, approximately 50–100 m from the Kenan stream. The majority of dwellings in the village were mud huts with thatched roofing. No artificial markers were used in the collection of these swarms. Swarms were collected on six consecutive nights and labelled Swarm #1–6, with Swarm #6 collected on the final day. The first three swarms collected were stored in 70% ethanol and the remaining three swarms were stored on silica gel.

### Species identification

All samples were morphologically identified using dichotomous keys [3]. Seven samples were too damaged and morphological identification could not be confirmed.

DNA was extracted using a DNA extraction kit prepGEM^®^Insect (CAT No.: PIN0050). Members of the *An. funestus* group were molecularly identified to species level by using a standard multiplex PCR [14], while the morphologically unknown samples were also analysed using the multiplex *An. gambiae* PCR [27]. Samples that were identified as “*An*. *gambiae*” were further subjected to RFLP analysis using *Hha*l FastDigest (Thermo Scientific FastDigest, cat no: (#FD1854) to differentiate between *An. gambiae s.s.* and *Anopheles coluzzii* [28, 29].

### *Anopheles funestus* genotyping

The *An. funestus s.s.* samples were subjected to an RFLP assay for the identification of molecular type (M-, W-, or MW-type) [7, 30]. Briefly, the D3 region in the 28S ribosomal DNA region are amplified [30] and subjected to RFLP using the restriction enzyme *Hpa*II prior electrophoresis as described by Garros et al. [7].

### Clade characterization assay

The protocol described by Choi et al. [10] was used and three positive clade I and three positive clade II controls were added, as well as a “no DNA” negative control. The COI region are amplified and clade-specific probes as described by Choi et al. [10] were used to differentiate between clade I and clade II.

### Genitalia rotation observations

Dahan and Koekemoer [20] described five stages (S_0_–S_4_) during the rotation event where S_0_ is characterized by no rotation of the genitalia, S_1_: rotation is between 1° and 45°, S_2_: rotation is between 45° and 90° rotation, S_3_: rotation is between 90° and 135° and at S_4_ rotation is between 135° and 180° and has been completed. Each individual male mosquito was viewed under a microscope and the stage of genitalia rotation was recorded according to the criteria described in Dahan and Koekemoer [20].

### Spermathecae dissection

All females had their spermathecae dissected to determine insemination rate [31].

## Results

**Fig. 1:**
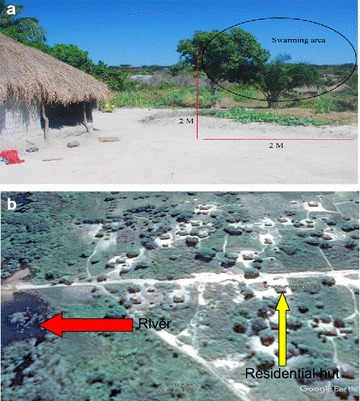
**a** Image of swarming location from ground view. The swarming area was in an area of 2 m by 2 m, above a white/grayish sandy area which had a defined break into green vegetated ground (inside black oval). Red vertical lines indicate height of swarm, horizontal line indicate the width of the swarming area. **b** Birds eye view of swarming location. Swarming location marked by pin with GPS co-ordinates (S09°19′28.6″ E028°47′06.9), red arrow marks putative breeding site, approximately 50–100 m away from swarming site. Yellow arrow marks residential hut

A total of six swarms were collected from a single swarming site. The swarming site was 5–10 m away from a residential hut and swarms were observed 2–2.5 m above the ground (Fig. 1). The swarms reformed each night at the same location and during the same period. The earliest swarming activity that was observed was at 18:05 and the latest was at 18:27 pm. However, swarming activity could still have been taking place thereafter but it was not possible to observe due to insufficient ambient light. The swarms collected were on average between 20 and 30 mosquitoes per swarm and is only an estimation based on size of the swarm and number of adults collected.

A total of 89 adult mosquitos were collected and amongst these 81 were males and 8 were females. Samples were morphologically separated into either *An. funestus* group (n = 82; 75♂ and 7♀) or unknown (if morphological identification was not clear; n = 7; 6♂ and 1♀). Of the 7 unknown groups, three were molecularly identified as *An. gambiae*. The remaining four samples (3♂ and 1♀) could not be identified to species level. All the *An. gambiae* complex samples collected were from one swarm (swarm #6) and were males. PCR followed by *Hha*I digest confirmed that they were *An. gambiae s.s.* (previously S-molecular form).

Females (n = 8) were collected in five of the six swarms, with most of the swarms (#1, #2, #3 and #6) containing a single female. Swarm #4 contained four females while females were not collected from swarm #5 (Table 1). Morphological analysis on the females showed that seven belonged to the *An. funestus* group and one female was too damaged to identify morphologically, but was still processed with the other samples. This sample failed to amplify for both the *An. funestus* and *An. gambiae* species-specific assays and remained unknown. The other females from the *An. funestus* group (n = 7) were identified as *An. funestus s.s.* and were all genotyped as the MW-type. Clade analysis demonstrated that the females belong to either clade I (n = 4) or clade II (n = 3) (Table 1). The presence of sperm in the spermatheca dissection confirmed that four of the females successfully mated.

**Table 1:** Identification and molecular typing of *Anopheles funestus* group collected in swarms in Nchelenge, Zambia

Swarm	Females					Males		
	Total samples collected	*An. funestus s.s.*	Mated	RFLP type	Clade I: Clade II^b^	Total number samples of *An. funestus* males	RFLP type	Clade I: Clade II^b^
1	1	1	1	MW	0:1	7	MW	5:2
2	1	1	–	MW	1:0	4	MW	4:0
3	1	1	1	MW	0:1	24	MW	18:5
4	4^a^	3	2	MW	3:0	19	MW	18:0
5	–	–	–	MW	0:0	7	MW	6:1
6	1	1	–	MW	0:1	12	MW	10:2
Total	8	7	4	–	7	73	73	71

^a^One female sample from this swarm was damaged beyond morphological identification and was molecularly identified as an unknown Anopheline

^b^Depicts the ratio of Clades I to II

**Fig. 2:**
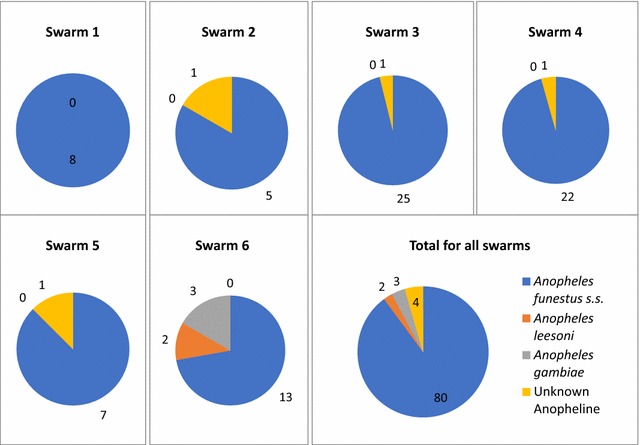
Shows the PCR identifications that were collected in each swarm

A total of 75 *An. funestus* group males were collected. Species-specific PCR identified two male *An. leesoni* from swarm #6, while the remaining 73 amplified as *An. funestus s.s.* (Fig. 2). These samples were further genotyped and belong to the MW-RFLP type (n = 73). Clade analysis identified both clade I (n = 61) and clade II (n = 10). Two samples failed to amplify during Clade analysis and this might have been due to inhibitors in DNA extraction. The majority of the swarms contained both clade types, except for swarm #2 (n = 4) and #4 (n = 19) which comprised of only clade I (Table 1).

The stages of genitalia rotation were analysed for all the swarming males regardless of species (Table 2). The majority (89%) of *An. funestus* males were classified as S_4_, and only two samples (2.74%) were S_3_. One *An. leesoni* male showed S_3_ rotation while the second sample was fully rotated (S_4_). Two *An. gambiae* samples were fully rotated (S_4_), while the one sample could not be analysed due to damage. Males from the “unknown anophelines” (n = 3) were also at S_4_.

**Table 2:** The frequency of genitalia rotation of swarming males

	*Anopheles funestus s.s.*	*Anopheles leesoni*	*Anopheles gambiae*	Unknown anophelines
Stage 3	2	1	–	–
Stage 4	65	1	2	3
Too badly damaged to evaluate rotation	6	–	1	–
Total	73	2	3	3

## Discussion

This study identified wild *An. funestus* group swarms in Nchelenge, Zambia and adult male were characterized in regards to the stage of genitalia rotation as well as species identification. Although observations were made in regards to height of swarms and relative location in regards to homes, this information is of limited value as swarming was only identified in one specific location. Six swarm collections were made from this location and this highlights the difficulty of finding *An. funestus* swarms in southern Africa. The observations made on the swarms in Nchelenge were consistent with that of Charlwood et al. [12] findings, confirming *An. funestus* swarmed 2–4 m off the ground. This is in contrast with Harper’s [13] observations that *An. funestus* swarms occur immediately inside the threshold of a hut, and swarming occurred a foot off the ground. The possibility that the swarm that Harper [13] studied was another member of the *An. funestus* group cannot be excluded, since molecular species identification was not available at that time. Another possibility is that *An. funestus* might change its swarming behaviour and position depending on environmental conditions. No swarming was observed on the thresholds of huts during this study in Zambia, but future studies in other countries and localities should be investigated to address this variation.

Swarms were observed close to a stream as was found by Charlwood et al. [12] who, like others [26, 32], noted that members of the *An. gambiae* complex swarm over areas of contrast on the ground, but *An. funestus* appears to avoid ground markers (both light and dark). Observations in Nchelenge indicated that swarming occurred at the transition point between bare ground (light colour) and low lying vegetation (dark colour) which have acted as a horizontal contrasting marker. Charlwood et al. [12] reasoned that *An. gambiae* complex and *An. funestus* swarms differentiate from each other by the markers over which they swarm.

All the adults were collected from the swarm every night, this did not appear to prevent a swarm forming on consecutive nights. Although the reason for this is difficult to speculate about, it is most likely that the swarm was replenished by emerged males that have subsequently matured and were therefore now ready for mating. Whether this will also occur in other parts of Africa is still to be determined. It is also unknown if *An. funestus* swarm in multiple small swarms or in fewer large swarms. Swarm numbers reported by Charlwood et al. [12] were also relatively low and on average less than 50 adults/swarm were recorded. Harper [13] estimated 300–500 mosquitoes were present in the swarm that he reported on.

All the *An. funestus* group samples were molecularly identified as *An. funestus* from the MW-type. Previous work identified MW-type from one sample from Chipata (bordering Malawi) while 33% (2/6 samples) from Ndola (close to the Democratic Republic of Congo) also carried the MW-type [8]. The current field site, Nchelenge, is approximately 500 km North of Ndola and this is the first samples that have been analysed from this specific area. Mitochondrial analysis revealed that both clades I and II occurred in the four of the six swarms. The ratio of clade I (86%): clade II (14%) is similar as to that recorded in the wild adult population 80% clade I and 20% clade II [11]. The sample size (n = 71) is however small and this might explain the shift towards clade 1. To date there has been no evidence to suggest that mitochondrial subpopulations are resulting in population isolation. Michel et al. [9] postulated that clade II originate from historical gene flow between previously isolated populations or even related species. In addition to this, the fact that adults of both clade types were found in the same swarm implies that no specific clade swarming characteristics were identified during this study. Since sperm from mated females were not analysed as part of this study, it is unknown if there is population structuring maintained through mating.

Only two samples of another member of the *An. funestus* group, *An. leesoni,* were collected, showing that mixed swarms can occur, although in a small proportion (less than 3%). *Anopheles leesoni* have been reported from this study site and a relatively small number of *An. leesoni* have been collected indoors (Venter, unpublished), but is known to occur in Zambia [2]. In addition to this, three *An. gambiae s.s.* samples were also collected, although it should be highlighted here that since morphological identification was not possible, their molecular identification cannot be guaranteed. The mixed species swarms were recorded during two swarm collection nights. Sweep nets were cleared after each collection to prevent contamination between collections. Charlwood et al. [12] also collected individual *An. gambiae s.l* samples amongst the *An. funestus* group swarms.

Mixed species swarms have been recorded by other authors and are characterized by always having only a few samples of the mixed species (low frequency). For example, Sawadogo et al. [29] reported mixed swarms between *An. gambiae s.s* and *An. coluzzii* in Burkina Faso, but this was not the case in Mali [29]. Sawadogo et al. [29] showed that *An. gambiae s.s* swarm earlier to compared to *An. coluzzii*, and although the swarming times seldom overlapped even if the same marker were used, environmental factors from time to time can result in few late swarming *An. gambiae* males to overlap with those of *An. coluzzii* swarming and vice versa, and hence result in the low frequency of mixed swarms. Diabaté et al. [32] showed that although the frequency of mixed swarms between *An. gambiae s.s* and *An. coluzzii* is low, it is still higher than the frequency of hybridization. They, therefore, concluded that mate recognition is more important than swarm segregation. It is still unclear if swarm collection on six consecutive times resulted in artificially interfering with swarms and hence resulted in mixed swarms (n = 2 *An. leesoni*) collected on the 6th day.

Due to the absence of knowledge of swarming behaviour of members of the *An. funestus* group (including *An. funestus*) it is not possible to postulate on the mixed swarms identified here. However, this emphasizes the need for additional research in this regard. Regardless of species, most of the males showed fully rotated claspers further indicating that immature males do not have the tendency to swarm. Future studies should also identify the mitochondrial type of the sperm in the mated females.

## Conclusion

In conclusion, although this study is only providing information on *An. funestus* swarming from one location in Zambia, it is the first report to molecularly characterize swarms from the main African malaria vector *An. funestus*. The results suggest that when the *An. funestus* male matures physiologically, there are subsequent behavioural changes which may trigger swarming behaviour. Although this study has provided additional insight into the composition of *An*. *funestus* swarms, additional studies should be initiated to characterize swarm height, location in respect to human dwellings as well as swarm markers in other African countries. Furthermore, it will be important to evaluate these swarm characteristics between other members of the *An. funestus* group and obtain a better understanding in regards to the composition of these swarms. This paper highlights the importance of instigating future studies in regards to swarming of this species group.

## Abbreviations

PCR: polymerase chain reaction
RFLP: restriction fragment length polymorphism
M-type: Malagasy and East African sequence
W-type: West central African sequence
MW-Type: Southern African sequence
